# Supplementary material for: No pets allowed: Evidence that prolonged grief disorder can occur following the death of a pet
Source: PLoS One. 2026 Jan 14;21(1):e0339213. doi: 10.1371/journal.pone.0339213 (PMC12803462; doi:10.1371/journal.pone.0339213)
Supplement: Attachment — Submitted filename: renamed_95a88.docx [file pone.0339213.s001.docx]

**CONSIDERATIONS:**

- The subject is treated in a complete way; the article is solid.
- It has a scientific interest, complements the existing information in literature, the original contributions are well achieved.
- The results are well elaborated, the discussion and conclusions are strong, their contribution is important to the existing literature.
- There is overall coherence in the development of the article.
- The references are written in Vancouver style; however, I did not find the referencing in the text, the bibliography is not listed.
- The wording is clear and precise.
- The figures and tables are understandable, I suggest reworking figure 1, it looks pixelated.
- The methodology is well constructed, a remark is made in the "notes" section.
- The discussion is good and well structured, clinically relevant issues are discussed.
- It is a novel and necessary issue to generate an update of the definition of PGD in future consensus.

**NOTES:**

- Page 8: Scores ≥ to… (¿?), incomplete information.
- The study uses the cultural criterion of PGD (symptoms that exceed what is normal in the participant's social/cultural/religious context). It is noted in a footnote that a strict reading of ICD-11 could exclude cases that answered, "I don't know" (16.6%, n=137), but that previous studies followed that only excluded those that answered "No". The rationale for this decision (including n=137) should be moved to the methods section and discussed further as a limitation or a key methodological decision. The impact of strict exclusion of these cases on PGD rates should be briefly considered in the Discussion.
- I suggest mentioning as a limitation the fact that the selection of participants from online research panels (Qualtrics) could bias the results (for example, towards those with greater digital access or certain demographic trends not covered by quotas).
